# Curing Kinetics Modeling of Epoxy Modified by Fully Vulcanized Elastomer Nanoparticles Using Rheometry Method

**DOI:** 10.3390/molecules27092870

**Published:** 2022-04-30

**Authors:** Mohammad Hossein Karami, Mohammad Reza Kalaee, Saeideh Mazinani, Mohamadreza Shakiba, Saied Shafiei Navid, Majid Abdouss, Alireza Beig Mohammadi, Weisong Zhao, Mojtaba Koosha, Ziyue Song, Tianduo Li

**Affiliations:** 1 Nanotechnology Research Centre, South Tehran Branch, Islamic Azad University, Tehran P.O. Box 19585-466, Iran; karami.polymerphd@gmail.com; 2 Department of Chemical and Polymer Engineering, South Tehran Branch, Islamic Azad University, Tehran P.O. Box 19585-466, Iran; 3 New Technologies Research Center (NTRC), Amirkabir University of Technology, 424 Hafez Ave., Tehran P.O. Box 15875-4413, Iran; s.mazinani@aut.ac.ir; 4 Department of Chemistry, Amirkabir University of Technology, Tehran P.O. Box 15875-4413, Iran; rezashakiba011@gmail.com (M.S.); phdabdouss44@aut.ac.ir (M.A.); ar.beigmohammadi@gmail.com (A.B.M.); 5 Faculty of Chemistry, University of Mazandaran, Babolsar P.O. Box 95447-47416, Iran; s.shafiei@stu.umz.ac.ir; 6 Shandong Provincial Key Laboratory of Molecular Engineering, School of Chemistry and Chemical Engineering, Qilu University of Technology (Shandong Academy of Sciences), Jinan 250353, China; 17854119208@163.com; 7 Department of Chemical and Biological Engineering, University of British Columbia, Vancouver, BC V6T 1Z4, Canada; songziyueca@outlook.com

**Keywords:** elastomer nanoparticles, epoxy resin, rheometer, gel time, chemorheology, curing kinetics model

## Abstract

In this study, the curing kinetics of epoxy nanocomposites containing ultra-fine full-vulcanized acrylonitrile butadiene rubber nanoparticles (UFNBRP) at different concentrations of 0, 0.5, 1 and 1.5 wt.% was investigated. In addition, the effect of curing temperatures was studied based on the rheological method under isothermal conditions. The epoxy resin/UFNBRP nanocomposites were characterized via Fourier transform infrared spectroscopy (FTIR). FTIR analysis exhibited the successful preparation of epoxy resin/UFNBRP, due to the existence of the UFNBRP characteristic peaks in the final product spectrum. The morphological structure of the epoxy resin/UFNBRP nanocomposites was investigated by both field emission scanning electron microscopy (FESEM) and transmission electron microscopy (TEM) studies. The FESEM and TEM studies showed UFNBRP had a spherical structure and was well dispersed in epoxy resin. The chemorheological analysis showed that due to the interactions between UFNBRP and epoxy resin, by increasing UFNBRP concentration at a constant temperature (65, 70 and 75 °C), the curing rate decreases at the gel point. Furthermore, both the curing kinetics modeling and chemorheological analysis demonstrated that the incorporation of 0.5% UFNBRP in epoxy resin matrix reduces the activation energy. The curing kinetic of epoxy resin/UFNBRP nanocomposite was best fitted with the Sestak–Berggren autocatalytic model.

## 1. Introduction

Among thermosetting polymers, epoxy resins are one the most notable thermosets due to their distinguished electrical, chemical and mechanical properties [1,2]. Epoxy resins prevalently used as matrices in reinforced composites, adhesives, surface coatings and the automotive industry [3,4,5,6].

One of the most practical methods in the polymer industry, especially in epoxy resins, is enhancing the toughening of polymers through the addition of nanoparticles [7,8,9]. Elastomeric nanoparticles could increase the toughness as well as the thermal characteristics of epoxy resins [10,11,12,13,14,15]. Ultra-fine full-vulcanized acrylonitrile butadiene rubber (UFNBRP) as an elastomeric nanoparticle is a new type of toughness modifier in polymer matrix composites that have recently gained great attention in industry. UFNBRP as an additive have been used in various polymers such as polypropylene (PP), polyvinyl chloride (PVC), polybutylene terephthalate (PBT), polylactic acid (PLA) and epoxy resins in order to modify the physical and mechanical properties of the resulting products. Zhao et al. investigated the toughening of PLA with UFNBRP. It was found that adding 20 wt.% UFNBRP improved the tensile strength due to the proper interaction between elastomeric nanoparticles and PLA [16]. Wu et al. showed that adding 20 wt.% of elastomeric nanoparticles to PBT improved their impact strength [17]. The physical, mechanical and thermal properties of epoxy coatings, including UFNBRP, were investigated in our previous research works [18,19,20]. The results showed that increasing UFNBRP improves the mechanical properties.

The incorporation of UFNBRP in epoxy resin matrix can affect the curing kinetics of the system. Understanding the curing behavior of epoxy resins is essential to achieve any enhancement in their curing and in the properties of the final product [21]. The rate of the reaction is the governing factor in controlling the epoxy curing process, thus accurate curing kinetic analysis might be a practical method for industrial applications [22,23,24]. As a result, having a precise understanding of the required time for the entire or partial curing reaction at a certain temperature in order to achieve the expected properties would be beneficial. Curing agents or hardeners for thermosets are crucial components and play a vital role that affect the properties and applications of the final product [6,15].

The rheological behavior plays an important role in controlling the curing process, which leads to improving the quality of the final product [25,26,27]. One of the advantages of the rheological measurements is that they can directly show the storage modulus variations like elasticity in the curing process, which is linked to the physical and chemical properties of the final product [28,29,30].

In a previous work by our team, the rheological curing kinetics of nanoclay/vinyl ester resin nanocomposites were studied. The results showed that, by increasing the curing temperature, the addition of clay nanoparticles decreased the curing rate, as well as the curing time [26]. The curing kinetics of cyanate ester resin/graphene oxide (GO) nanoparticles was investigated in another study. It was observed that, in non-isothermal conditions, adding GO nanoparticles had a strong catalytic effect on the curing process, especially in the early stages. The results also showed that the most effective catalytic behavior was observed in 1 wt.% GO [25]. In another study, the effect of calcium carbonate (CaCO_3_) nanoparticles in the curing kinetics of polyester/epoxy resin was investigated. The results exhibited that adding 5 wt.% of CaCO_3_ decreases the activation energy (E_a_) and the curing rate [27]. The curing kinetics of epoxy nanocomposites including nanoclay and diaminodiphenyl methane (DDM) nanoparticles was studied by another work. The results showed that nanoclay particles has no effect on the curing kinetics, through iso-conversional modeling. However, it was shown that adding nanoclay particles has a huge effect on activation energy (E_a_) during the curing process [31].

According to our previous studies, the physical and mechanical properties of epoxy resins are affected by elastomeric nanoparticles [20]. However, achieving the final properties of epoxy resins modified by elastomeric nanoparticles depends on the curing kinetics of the system. To the best of our knowledge, the study of epoxy curing kinetics in the presence of UFNBRP elastomeric nanoparticles has not been studied.

Herein, the epoxy/UFNBRP nanocomposites were prepared successfully by sonication process. The morphology of the nanocomposites was studied using scanning electron microscopy (SEM) and transmission electron microscopy (TEM). The isothermal rheological experiments at different temperatures (65, 70 and 75 °C) and different amounts of UFNBRP (0.5, 1 and 1.5 wt.%) were used to investigate the curing kinetics of epoxy/UFNBRP nanocomposites. To choose the right model for the reaction rate and E_a_, different models were investigated and, finally, the Sestak–Berggren autocatalytic model showed the best fit on the experimental data of the nanocomposites. This work has novelty for academic and technological purposes, because the curing kinetics of epoxy/UFNBRP nanocomposites is still an open window for new achievements and research. Practically, most epoxy resin nanocomposites are processed by thermal curing, so the reported curing kinetics data of this work can be useful for industrial applications to set the optimum time and temperature of the curing process.

## 2. Materials and Methods

### 2.1. Material

Huntsman Araldite LY564 epoxy resin with Huntsman Aradur 2954 amine hardener were supplied by Huntsman Co. (Salt Lake, UT, USA). The NBR nanoparticles were obtained from SINOPEC Beijing (Beijing, China) research institute. NBR nanoparticles size is smaller than 100 nm with narrow distribution and fully of vulcanization.

### 2.2. Sample Preparation

**Figure 1:**
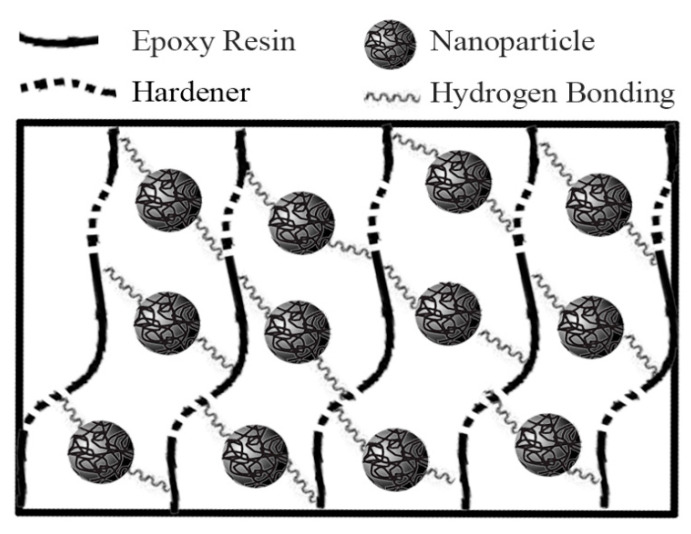
An illustration of the epoxy resin/UFNBRP nanocomposite structure.

A certain amount (0.5, 1 and 1.5 wt.%) of UFNBRP was pre-mixed with epoxy resin at a mixing rate of 2000 rpm to obtain the optimum mixing condition. After approximately 20 min, the solution was completely sonicated and mixed again with a homogenizer for 20 min at 10,000 rpm. After adding the hardener, the mixing was continued for 2 min at 2000 rpm. The mixture was vacuum-dried at environment temperature for 1 h before mixing and 2 h after mixing to obtain bubble-free samples. Afterwards, the samples were immediately used for the rheological test. The illustration of the structure of epoxy resin intercalated with UFNBRP is shown in Figure 1.

### 2.3. Instrumental Analysis

Scanning electron microscopy (SEM) images were captured from the fractured surfaces in nitrogen using a TESCAN (Vega III, Brno-Kohoutovice, Czech Republic), and transmission electron microscopy (sliced specimens from the core) was used for morphological study using PHILIPS, TECNAI20, The Netherlands. Rheological studies carried out on the MCR300, Anton Paar rheometer (Graz, Austria), tests were performed at 65, 70 and 75 °C under isothermal conditions at 1 Hz frequency. The viscoelastic range was considered linear to record viscoelastic functions and to calculate the curing kinetics of the crosslinking at isothermal conditions.

### 2.4. Cure Kinetics Analysis

Mathematical models can be used for the analysis of the degradation or curing kinetics of thermoplastic or thermoset polymers [32]. The curing kinetics of thermoset polymers is defined as Equation (1) [33]:

(1) $$\frac{d\alpha }{\mathrm{dt}}=k\left(T\right)f\left(\alpha \right)$$

where α is the degree of curing reaction, and f(α) is a function related to the type of kinetic model. k(T) is the constant related to the reaction rate, as expressed in Equation (2) according to the Arrhenius model:

(2) $$K\left(T\right)=\mathrm{Aexp}\left(-\frac{E_{a}}{\mathrm{RT}}\right)\;$$

where E_a_ is the activation energy in $\left(\frac{\mathrm{kJ}}{\mathrm{mol}}\right)$, T in Kelvin and R is the universal gas constant.

Table 1 demonstrates the regression parameter calculated from some commonly used kinetic models based on Equations (1) and (2) [34,35,36,37]:

## 3. Result and Discussion

### 3.1. Nanocomposites FTIR Study

**Figure 2:**
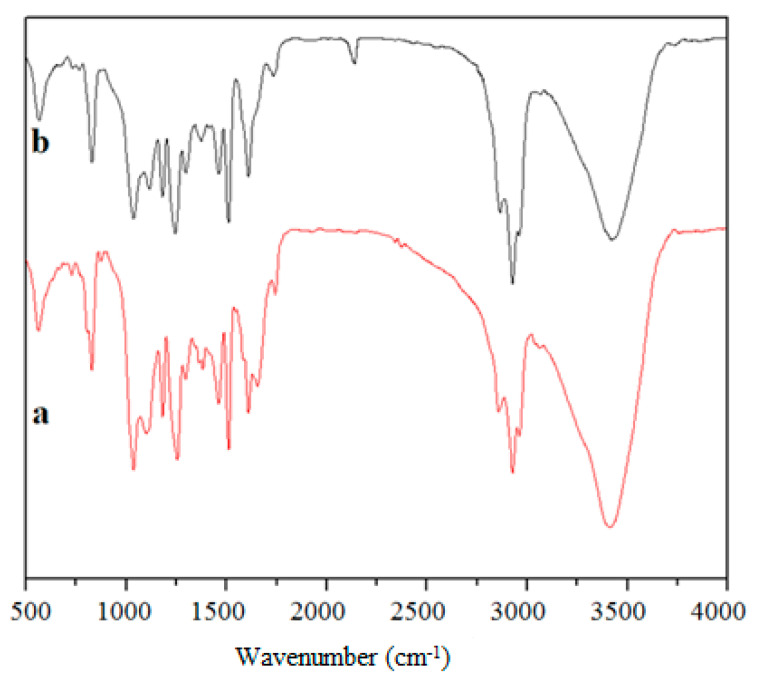
FTIR spectra of (**a**) neat epoxy resin and (**b**) epoxy/UFNBRP nanocomposite.

To investigate the interaction between the epoxy resin and UFNBRP, the FTIR technique images have been used, and the images are given in Figure 2. In the FTIR spectrum of neat epoxy resin (Figure 2a), the broad absorption band at 3419 cm^−1^ related to OH stretching vibration. The absorption bands at 2862–3060 cm^−1^ are related to the stretching vibration of aromatic CH and CH (CH_2_ and CH_3_) in aliphatic groups in the epoxy resin structure. The multiple stretching for C=C vibrations in aromatic rings appeared at 1509–1734 cm^−1^. The absorption bands related to the bending vibration of aliphatic groups can be found at 1244–1373 cm^−1^, and the absorption bands at 1036–1180 cm^−1^ are related to the C–O and C–O–C groups. The spectrum of epoxy resin is compatible with the literature [6,38]. In the FTIR spectrum of epoxy/UFNBRP (Figure 2b), the absorption band appearing at 2214 cm^−1^ is related to the CN group in the rubber structure. Because of the appropriate interaction between the epoxy resin and UFNBRP, the intensity of the OH group was decreased, which revealed an interaction between CN and OH groups. As can be seen, there is no other obvious change in absorption bands in the FTIR spectrum of epoxy/UFNBRP compared to the neat epoxy resin, which could possibly be due to the physical interaction between the UFNBRP and the epoxy. In fact, UFNBRP and epoxy are available as a blend.

### 3.2. Morphology of Nanocomposites

**Figure 3:**
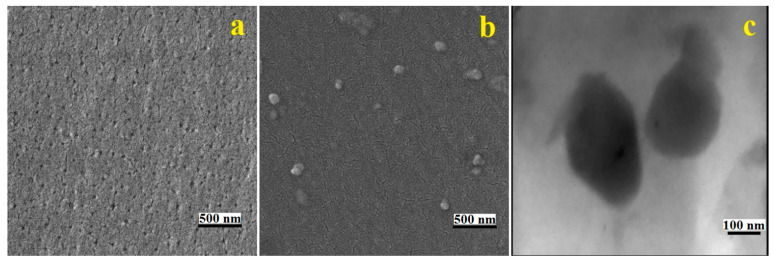
SEM images of (**a**) neat epoxy resin and (**b**) epoxy resin/1% UFNBRP, and TEM image of (**c**) epoxy resin/1% UFNBRP.

The SEM images of neat epoxy and the epoxy resin/1% UFNBRP nanocomposite at 50 kx magnifications and the TEM image of epoxy resin/1% UFNBRP are given in Figure 3.

The SEM analysis shows that the UFNBRP is uniformly dispersed in epoxy resins, due to the great interface and intense interactions, including hydrogen bonding and chemical reaction on the interface between UFNBRP and epoxy resin [38]. The epoxy resin composite modified by UFNBRP has a large interface because of the large specific surface area of nanoparticles, it has also been found that the uniform dispersion of elastomer nanoparticles can be certified by sufficient blending time and proper UFNBRP composition [12,17,39]. According to the TEM analysis, the morphology of the elastomeric nanoparticles is spherical, and there is a proper dispersion in each sample containing the elastomeric nanoparticles. Due to the higher resolution of the TEM images, the results acquired from SEM images are confirmed to be related with the size and morphology of the nanoparticles.

### 3.3. Reaction Kinetics by Rheometry (Chemorheology Analysis)

**Figure 4:**
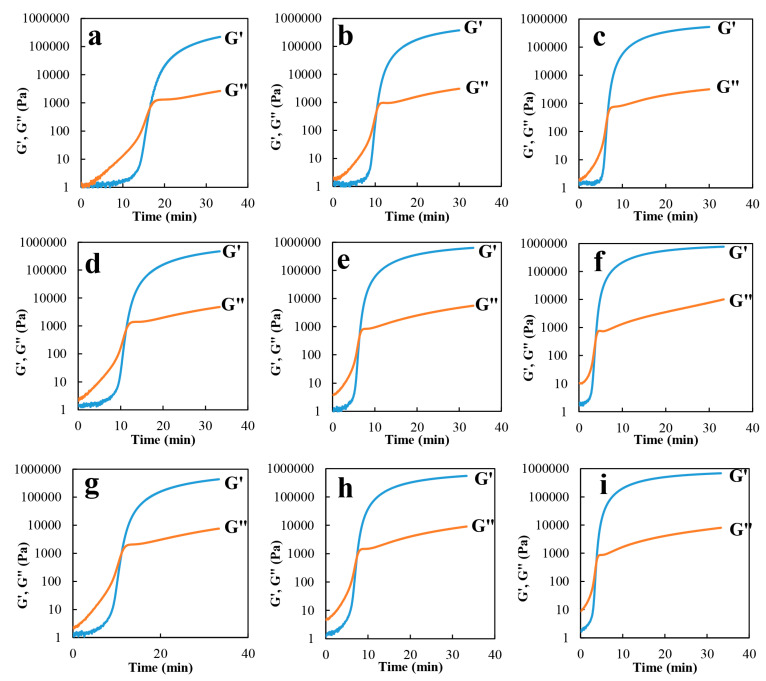
Rheological diagrams of epoxy resin/0.5% UFNBRP at (**a**) 65 °C, (**b**) 70 °C and (**c**) 75 °C; rheological diagrams of epoxy resin/1% UFNBRP at (**d**) 65 °C, (**e**) 70 °C and (**f**) 75 °C; rheological diagrams of epoxy resin/1.5% UFNBRP at (**g**) 65 °C, (**h**) 70 °C and (**i**) 75 °C.

Chemorheology is the study of the change in viscoelastic properties in reacting systems, including an analysis of the alteration in viscosity as a function of the chemical reaction’s conversion [40,41]. The effect of adding UFNBRP on rheological characteristics of epoxy resin with 0.5%, 1 and 1.5% of UFNBRP at different temperatures, 65, 70 and 75 °C, are presented in Figure 4. The intersection of G″ and G′, known as gel point ($t_{g}$), was used for the subsequent calculations of chemorheology measurements.

As shown in Figure 4, at a constant temperature of 65, 70 and 75 °C, the curing rate decreases as UFNBRP composition increase at gel point (gel time), due to the interactions between UFNBRP and the curing agents with epoxy resin. According to previous studies, more hydrogen bonds formed between the nitrile group of UFNBRP and the hydroxyl group of epoxy resin [11].

The gel point is usually determined as the time or the degree of cure at which the solution no longer remains liquid. Originally, qualitative rheological investigation was used to define the gel point. The intercept of loss modulus and storage modulus can be considered the gel point (Figure 4). The critical region starts with an abrupt increase in G′, and when G′ and G″ intercept, tan δ = 1. After intercepting, G′ is higher than G″ and tan δ becomes smaller than 1 [42,43,44]. The gel times of the sample were taken as the time at the cross-over point of storage and loss modulus and demonstrate the point at which the material changes from a liquid to a solid [45]. The same results were observed for different epoxy nanocomposites, which shows that the increasing of different concentrations of UFNBRP had no impact on the gel point formation trend [19].

### 3.4. Effect of Elastomeric Nanoparticles on the Complex Viscosity at Different Temperatures

**Figure 5:**
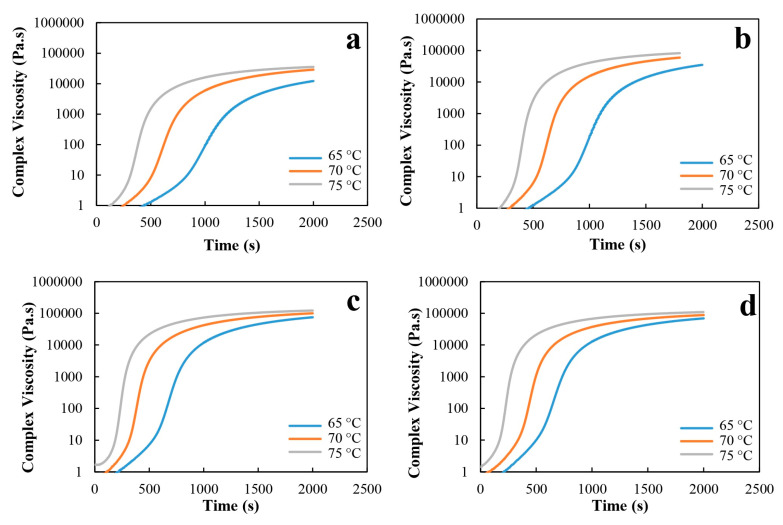
The complex viscosity versus time at different temperatures of 65, 70 and 75 °C for (**a**) epoxy resin, (**b**) epoxy resin/0.5% UFNBRP (**c**) epoxy resin/1% UFNBRP and (**d**) epoxy resin/1.5% UFNBRP.

Figure 5 shows the complex viscosity versus time for neat epoxy resin and nanocomposites at temperatures of 65, 70 and 75 °C. As can be observed, as the temperature reaches the curing time, the complex viscosity increase, which is the beginning of the crosslinking process. Moreover, in Figure 5, the curve does not show any particular change, since it is associated with the kinetics change during the process, especially before the gel point [46]. In the epoxy resin and epoxy nanocomposite samples, at first the complex viscosity is constant, and as time passes, the viscosity increases [47]. It is clear from Figure 5 that, as the curing temperature was increased, the gel point time decreased and the curing rate accelerated. At the beginning of the curing reaction, viscosity is constant with time, and at a certain point, it exhibits a very fast increase. This increase in complex viscosity curves is interpreted to happen in a shorter time with UFNBRP, because of the reaction between UFNBRP and epoxy resin. The cure-accelerating capability of the UFNBRP provides earlier crosslink formation and faster achievement of the gel point [48,49,50].

### 3.5. Evaluation of Gel Point and Activation Energy (Ea) Calculation by Rheometry

**Figure 6:**
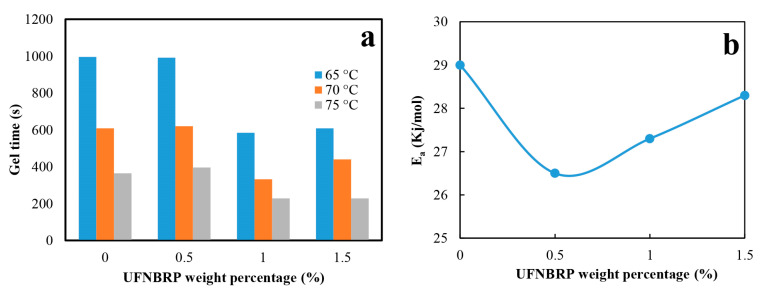
(**a**) Gel time diagram for the epoxy resin and epoxy resin/UFNBRP nanocomposites and (**b**) activation energy diagram of neat epoxy resin at different elastomeric nanoparticles concentrations.

At the gel point, the viscosity of epoxy resin increases sharply and the rate of reaction is controlled by the penetration phenomenon, while the resin processability is reduced. Glassing usually occurs after the gel point, when the degree of molecular weight increases and further crosslinking reduces the degree of crosslink [51]. Many researchers calculate the gel point where the storage modulus intercepts the loss modulus. In crosslinking reactions that are being studied by rheological method, first loss modulus and storage modules start to rise, before the gel point, the system exhibits viscous behavior (G″ > G′); at gel point (t_g_), they are equal (G″ = G′) and the system shows elastic behavior (G′ > G″) [52]. The gel point was used to study the kinetics of curing for the above nanocomposites. Considering this method, the activation energy of curing is calculated using Arrhenius Equations (3) and (4) as follows:

(3) $$t_{g}=\frac{1}{K\left(T\right)}\int_{0}^{\alpha \;\mathrm{gel}}\cdot \frac{1}{g\left(\alpha \right)}\;d\alpha$$

(4) $$K\left(T\right)=\mathrm{Aexp}\left(-\frac{\mathrm{Ea}}{\mathrm{RT}}\right)$$

where $t_{g}\;$ is the gel time (s), $E_{a}$ is the activation energy in $\left(\frac{\mathrm{kJ}}{\mathrm{mol}}\right)$, T is temperature in Kelvin, and R is the universal gas constant. The calculation results for E_a_ of curing epoxy/UFNBRP are presented in Figure 6b.

As could be observed in Figure 6b, the E_a_ changed upon UFNBRP addition, as well as with temperature. Ea of neat epoxy is 29.00 $\left(\frac{kJ}{mol}\right)$ and with the addition of 0.5% UFNBRP Ea decreased to 26.48 $\left(\frac{\mathrm{kJ}}{\mathrm{mol}}\right)$. Furthermore, with 1% and 1.5% of UFNBRP, Ea increased to 27.21 $\left(\frac{\mathrm{kJ}}{\mathrm{mol}}\right)$ and 28.10 $\left(\frac{\mathrm{kJ}}{\mathrm{mol}}\right)$, respectively. It can be clearly understood that, for the samples with the same UFNBRP composition, the onset of curing and curing time decreases as the temperature increases. This might be attributed to the availability of more thermal energy for the curing reaction at higher curing temperatures [53].

Onset of cure and $G_{\infty }^{\prime }$ are presented in Table 2. The data show that, by increasing the temperature, onset of cure occurs in shorter times and $G_{\infty }^{\prime }$ increases. The addition of UFNBR also has a similar effect by comparing these two parameters between the samples.

### 3.6. Effect of Elastomeric Nanoparticles on Degree of Curing at Constant Temperatures

The degree of curing (α), is defined according to Equation (5) where G′(t) is known as the storage modulus at any time, G′(0) is the modulus at the initial time, and G′(∞) is named as the storage modulus at infinite time:

(5) $$\alpha =\frac{G^{\prime }\left(t\right)-\;G^{\prime }\left(0\right)}{G^{\prime }\left(\infty \right)-\;G^{\prime }\left(0\right)}\;$$

**Figure 7:**
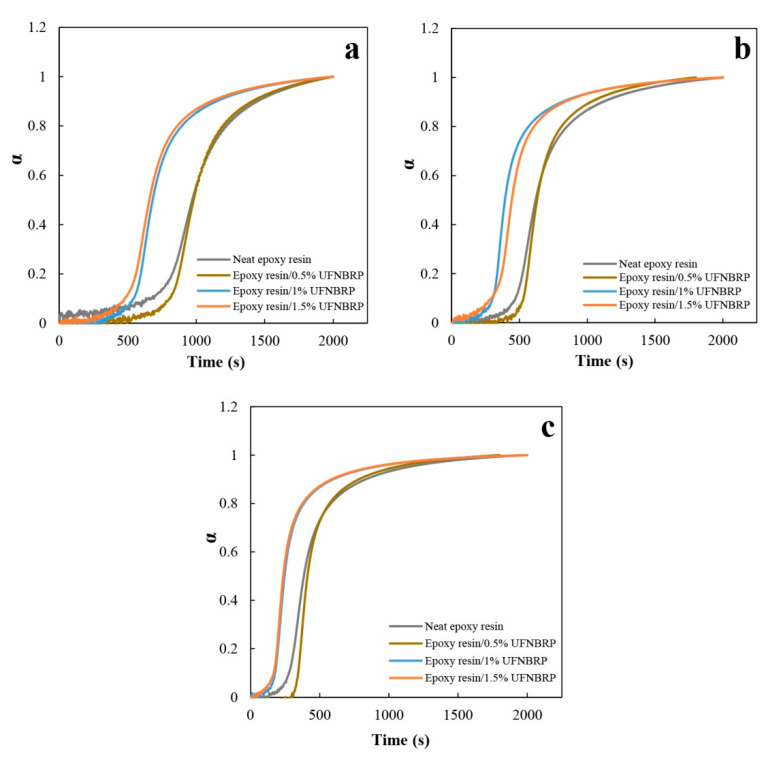
Degree of curing of epoxy resin and epoxy resin/UFNBRP nanocomposites at (**a**) 65 °C, (**b**) 70 °C and (**c**) 75 °C.

Figure 7 shows the changes in degree of curing versus time for neat epoxy resin and samples containing nanoparticles at temperatures of 65, 70 and 75 °C.

As shown in Figure 7, a very similar degree of curing versus time profiles was obtained for all investigated systems. The differences are more seen at higher temperature and at the initial stage of curing [54]. Adding UFNBRP reduces the curing time for samples containing 1 and 1.5% of UFNBRP, which could be related to the effect of the improved interaction between epoxy resin and the nanoparticles. The degree of conversion is constant before the onset of the cross-linking process and then increases massively with the curing time [30]. Moreover, for the epoxy resin and nanocomposites with 0.5% UFNBRP at the mentioned temperatures, there is no significant decrease in curing time. By increasing temperature from 65–75 °C, the curing time decreases. These presented plots in Figure 7 are called s-shaped curves and are sigmoidal. This indicates that the mechanism of the curing reaction is an autocatalytic mechanism [55,56,57].

### 3.7. Kinetics Analysis

Kinetics parameters of epoxy resins and epoxy/UFNBRP nanocomposites at temperatures of 65, 70 and 75 °C are shown in Table 3. The results show that the experimental data followed by curve-fitting could well obey Sestak–Bregrren, and the adjusted R-square statistic shows that experimental data obey the autocatalytic model to a great extent. With increasing amount of UFNBRP, the E_a_ initially decreased (until 1%), and then increased (from 1 to 1.5%). This is probably due to the accumulation of nanoparticles, which can also be seen in the SEM and TEM images. In general, E_a_ was lower in the epoxy/UFNBRP nanocomposites than in the neat resin [58,59,60].

**Figure 8:**
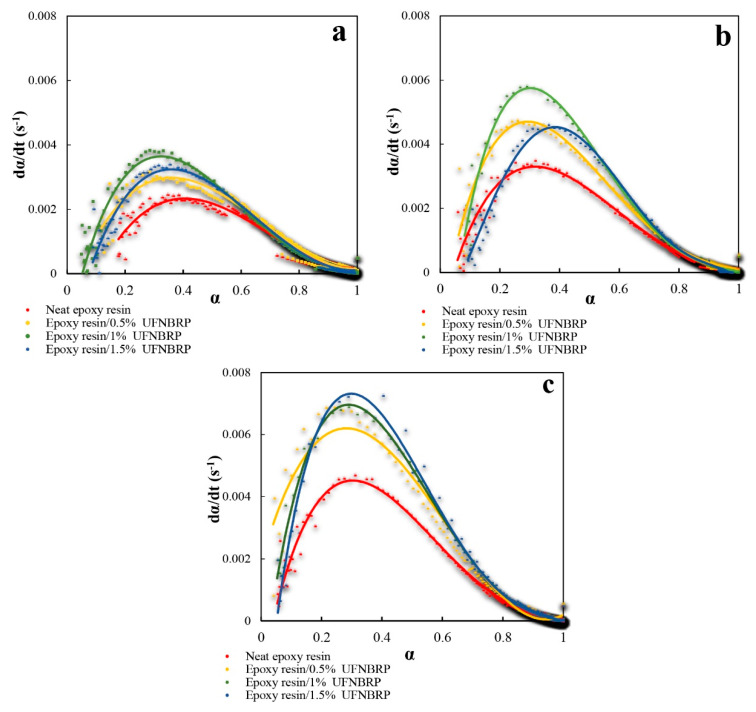
Variation in the curing rate of epoxy resin/UFNBRP nanocomposites at different temperatures of (**a**) 65 °C, (**b**) 70 °C, and (**c**) 75 °C.

The curing rate of epoxy resin and epoxy nanocomposites are depicted in Figure 8. As can be observed, the curing rate reaches a maximum value at a specified curing value, which could be known as a signal showing that the laboratory data follow the Sestak–Berggren autocatalytic model. We also have a slight deviation at low temperatures because of the large number of reactive groups’, which occurs at low conversion rates [61,62].

The final equations, which are predictive equations for the curing reaction, were obtained for neat epoxy and nanocomposites. The experimental results that were obtained from the reaction rate and the amounts achieved from the following relationships are given in Figure 8 in terms of the degree of curing for the samples [63]. Since the model line and the points obtained from the experiments are so close together, it is obvious that the Sestak–Berggren autocatalytic model is an appropriate curing kinetic model for characterizing neat epoxy resin and nanocomposites curing kinetics reaction. The curing kinetic equation of the epoxy resin and elastomeric nanoparticles obtained from the results of the model fitting was collected in Table 4.

## 4. Conclusions

The effect of elastomeric nanoparticles on the cure behavior of epoxy nanocomposites was studied using morphological and chemorheological methods and curing kinetics. The viscoelastic functions, $G^{\prime }$ and $G^{\prime \prime }$, were appraised isothermally at different temperatures (65 °C, 70 °C and 75 °C). The reaction rate was found, based on the Sestak–Berggren model. The morphological studies were performed by SEM and TEM images, and the results clearly demonstrated the suitable dispersion of nanoparticles in the epoxy matrix. The investigation of chemorheological methods and curing kinetics showed that, by adding UFNBRP, gel time is decreased and the viscosity of the complex increases in a short time, and also, for samples of 1% and 1.5% UFNBRP, the degree of curing decreases. It was demonstrated that the presence of UFNBRP in the epoxy resin system decreased the activation energy and increased the reaction order. The difference between these two methods is due to the mathematical root of the formula and also the dependence of the chemorheology method on gel time.

## Figures and Tables

**Table 1:** Common kinetic models for the cooking process.

Model	Kinetic Model (or theory)	R^2^
**nth-order**	$f\left(\alpha \right)=k\;\cdot {\left(1-\alpha \right)}^{n}$	Not fitted
**Sěsták-Berggren**	$f\left(\alpha \right)={k\alpha }^{m}\;\cdot {\left(1-\alpha \right)}^{n}$	0.95–0.97
**Horie**	$f\left(\alpha \right)=\left(k_{1}+k_{2}{\;\alpha }^{m}\right)\cdot {\left(1-\alpha \right)}^{n\;}$, m = 1	0.91–0.95
**Kamal**	$f\left(\alpha \right)=\left(k_{1}+k_{2}\alpha^{m}\right)\cdot {\left(1-\alpha \right)}^{n\;}$	Not fitted

where m and n are the kinetic parameters, and α is the degree of conversion. As can be observed, after fitting the experimental data to these models, Sestak-Breggren has the highest R^2^.

**Table 2:** Values of $G_{\infty }^{'}$, onset of cure and cure time for all of the samples at the analyzed temperatures.

Sample	Curing Temperature (^°^C)	$G_{\infty }^{\prime }\;(\mathrm{Pa})$	Onset of Cure (s)
Epoxy resin	65	76,800	1050
Epoxy resin	70	182,000	656
Epoxy resin	75	223,000	370
Epoxy resin/0.5%UFNBRP	65	221,000	1060
Epoxy resin/0.5%UFNBRP	70	375,000	692
Epoxy resin/0.5%UFNBRP	75	520,000	472
Epoxy resin/1%UFNBRP	65	471,000	728
Epoxy resin/1%UFNBRP	70	629,000	460
Epoxy resin/1%UFNBRP	75	768,000	300
Epoxy resin/1.5%UFNBRP	65	434,000	728
Epoxy resin/1.5%UFNBRP	70	553,000	524
Epoxy resin/1.5%UFNBRP	75	687,000	280

**Table 3:** Kinetics parameters of epoxy resins and epoxy resin/UFNBRP nanocomposites at temperatures of 65, 70 and 75 °C.

Sample	T (°C)	m	n	$k\left(S^{-1}\right)$	R^2^	$\left(\frac{\mathrm{kJ}}{\mathrm{mol}}\right)$ $E_{a}$
Epoxy resin	65	1.103	1.642	0.01252	0.90	139.80
Epoxy resin	70	1.258	2.373	0.03344	0.94	
Epoxy resin	75	1.282	2.607	0.0521	0.97	
Epoxy resin/0.5%UFNBRP	65	0.9885	1.784	0.01718	0.93	81.23
Epoxy resin/0.5%UFNBRP	70	1.045	2.297	0.03559	0.96	
Epoxy resin/0.5%UFNBRP	75	0.7977	2.348	0.02928	0.97	
Epoxy resin/1%UFNBRP	65	1.513	2.676	0.05607	0.92	22.72
Epoxy resin/1%UFNBRP	70	1.399	2.875	0.08207	0.97	
Epoxy resin/1%UFNBRP	75	1.167	2.624	0.07049	0.99	
Epoxy resin/1.5%UFNBRP	65	1.575	2.451	0.04728	0.96	114.67
Epoxy resin/1.5%UFNBRP	70	2.03	3.01	0.1323	0.96	
Epoxy resin/1.5%UFNBRP	75	1.565	3.139	0.1442	0.96	

**Table 4:** The predictive curing kinetics of Sestak-Berggren autocatalytic model.

Sample	Cure Kinetic Model
Neat epoxy resin	$\frac{d\alpha }{\mathrm{dt}}=0.1705\alpha^{\;1.214}\;{\left(1-\alpha \right)}^{2.207}$
Epoxy resin/0.5% UFNBRP	$\frac{d\alpha }{\mathrm{dt}}=0.0788\alpha^{\;0.944}\;{\left(1-\alpha \right)}^{1.548}$
Epoxy resin/1% UFNBRP	$\frac{d\alpha }{\mathrm{dt}}=0.0695\alpha^{\;1.359}\;{\left(1-\alpha \right)}^{2.725}$
Epoxy resin/1.5% UFNBRP	$\frac{d\alpha }{\mathrm{dt}}=0.1079\alpha^{\;1.198}\;{\left(1-\alpha \right)}^{2.866}$

## Data Availability

Not applicable.

## Sample Availability

Samples of the compounds are not available from the authors.
